# Solution-processed flexible neutron converter foils

**DOI:** 10.1038/s41598-026-69811-w

**Published:** 2026-09-05

**Authors:** Markus Köhli, Mikel Rincón Iglesias, Wen-Shan Zhang, Reshma Jolly, Jonas Marach, Jannis Weimar, Gerardo Hernandez-Sosa

**Affiliations:** 1 Physikalisches Institut, Heidelberg University, Im Neuenheimer Feld 226, 69120 Heidelberg, Germany; 2 HEiKA - Heidelberg Karlsruhe Strategic Partnership, Heidelberg University, Karlsruhe Institute of Technology (KIT), Heidelberg, Germany; 3 Light Technology Institute, Karlsruhe Institute of Technology - KIT, 76131 Karlsruhe, Germany; 4 BCMaterials,Basque Center for Materials, Bld. Martina Casiano, 48940 Leioa, Spain; 5 Organisch-Chemisches Institut, Heidelberg University, Im Neuenheimer Feld 270, 69120 Heidelberg, Germany; 6 Institute of Microstructure Technology, Karlsruhe Institute of Technology - KIT, Hermann-von-Helmholtz-Platz 1, 76344 Eggenstein-Leopoldshafen, Germany; 7 InnovationLab, Speyerer Str. 4, 69115 Heidelberg, Germany

**Keywords:** Neutron detector, Functional printing, Boron carbide, Lithium fluoride, Solution-processing, Cosmic-ray neutron sensing, Energy science and technology, Engineering, Materials science, Physics

## Abstract

The cost increase of helium-3 has sparked the development of alternative thermal neutron detection technologies, specifically the use of boron carbide converters is one of the pillars of next-generation detectors. While the state-of-the-art sputter-coating technique produces high-quality films, it is limited in deposition area, and requires costly and energy-intensive vacuum processing. Lithium fluoride can reach a similar performance to boron carbide, yet requiring thicker layers. The field of functional printing offers several advantages to improve cost efficiency: deposition possibilities over large areas, a larger palette of materials and mechanically flexible substrates. We investigated the deposition of boron carbide and lithium fluoride materials via bar coating to fabricate high-performance neutron-sensing flexible films. After establishing a theoretical model for detection efficiency, fabrication processes were successfully established, film properties characterized and their outgassing evaluated. Mechanically flexible coatings of boron carbide and lithium fluoride films were produced with layer heights between 2 µm and 50 µm and binder concentrations between 10 % and 30 % by weight. They show encouraging results in terms of performance and mechanical stability. Due to the industrial readiness of printing technologies we expect a potential pathway towards developing a new generation of neutron converter foils to support the development of state-of-the-art large-area instruments. Lower costs, more detectors, better research.

## Introduction

Agricultural practices are responsible for 70–80 % of the global freshwater consumption. The key to relieve such intensive water demand is improving resource usage by monitoring spatiotemporal soil moisture^1^ in managed irrigation systems which ensure that water reaches the crops in a timely manner^2^. To date, the tools for determining soil water content are either bound to point probes (e.g. dielectrical probes)^3^ or satellite-based products, often with kilometer-resolution^4^. Both approaches practically do not meet the spatial or temporal correlation lengths for soil moisture^5^. An emerging technology which enables smart farming through a transformation of water conservation strategies is Cosmic-Ray Neutron Sensing (CRNS)^6^. This non-invasive method is based on measuring the flux of a specific energy band of neutrons, produced in the upper atmosphere reflected from the soil. Neutrons in the epithermal to fast energy range (1 eV to 10$^5$ eV) are highly sensitive to hydrogen^7,8^, which turns neutron detectors into efficient proxies for changes in environmental water content. The sensors, mounted 1 to 2 meters above the ground, provide a count rate measurement representative of the average root-zone water content within a footprint of up to 18 hectares and 20 cm to 70 cm in depth^9,10^ or to the kilometer scale using the roving technique^11^. Thus, CRNS can assess and monitor the soil water deficit, which is critical for successful plant growth^12^. Recent developments have led to a broad success of the method due to its large footprint, low maintenance, and non-invasive nature. Such developments include using neutron converters^13^ other than the scarce $^3$He^14^. The CRNS system developed at Heidelberg University^15–17^ comprises neutron counting tubes utilizing a film of $^{10}$B enriched B$_4$C as a neutron-sensitive converter^18^. This layer has typically a thickness of up to 1.5 µm and is sputter-deposited on high purity copper foils. While producing high-quality films, sputtering is limited in the deposition area, and requires costly and energy-intensive vacuum processing^19,20^. Alternative low-temperature deposition approaches, such as hot-wire chemical vapor deposition, have also been investigated specifically for the fabrication of boron carbide neutron converter layers^21^. Therefore, new approaches for the fabrication of neutron sensing foils are necessary to improve fabrication costs, deposition over large areas and explore a larger palette of materials. The field of functional and flexible printing offers several advantages to face these challenges. It provides cost-efficient, low-temperature processing at ambient conditions with high throughput and large-area fabrication inherent to printing techniques. It also enables resource-efficient deposition of functional materials with low waste generation^22^. Current research in this field focuses on investigating new organic and inorganic materials and fully printable device architectures to fabricate photodetectors^23^, solar cells^24^, transistors^25^, light-emitting diodes^26^, and develop applications in wearable, communication, IoT fields, as well as in large area electronics^27^. At the industrial level, functional printing is an already established process for the metallization of photovoltaic devices and RF antennas^28^. In all these mentioned cases, however, it is necessary to tackle challenges in ink formulation and process optimization to yield not just a graphical printed pattern but a functional film with the intended (opto)electronic functionality. This can be addressed by various approaches such as solvent choice, forming composites, controlling substrate interactions, etc., depending on the material system and printing technology at hand^26,29,30^. Beyond environmental applications, there has been strong demand for thin neutron-absorbing coatings for instruments at large-scale research facilities (e.g., the European Spallation Source and the Chinese Spallation Source) for more than a decade^31^. These include detectors based on multi-wire proportional chambers^32–34^, resistive plate chambers^35^ and multi-layer structures using Gas Electron Multipliers^36,37^. In the field of homeland security replacement detectors have been introduced mainly based on boron-lined proportional chambers^38^. These type of detectors can require thin substrates, on which sputtering can cause significant problems due to the large strain forces by boron carbide layers^39^. Printing completely avoids these issues. For neutron-shielding applications^40^, in addition to hot rolling^41^ and cold-spray additive manufacturing^42,43^, recently fabricated thick, flexible boron carbide layers as MXene composite films. Additionally, thermal neutron shieldings which can be adapted to already assembled instruments can be produced by such^44^. Thick gadolinium-based paints have already been used, however, boron is considered more favorable due to the lower gamma background^45,46^. Finally, printing techniques also offer the possibility to combine layers of different neutron converters in order to increase the efficiency^47^.

The selection of materials plays a pivotal role in the fabrication of an efficient neutron detector. Only a few isotopes exhibit sufficiently high thermal absorption cross sections and produce charged-particle reaction products that are unambiguous. Table 1 summarizes the common converters and their primary reaction channels. We chose boron carbide (B$_4$C), boron metal (B), and lithium fluoride (LiF) for their neutron-absorption properties^48^. LiF^49^ can reach a similar performance with 20 µm layer thicknesses. While its lower melting point and lower costs are advantageous, there are currently no ideal film deposition techniques for this material. Printing techniques, however, can easily process this material, see also^50^. Polymethyl methacrylate (PMMA, C$_5$H$_8$O$_2$) and polyvinyl alcohol (PVA, C$_2$H$_4$O) served as binders embedding the converter and to form flexible, stable films. PMMA has been widely studied in the area of nano- and micro-composites due to its combination of high strength with thermal and dimensional stability. PVA is a synthetic biodegradable polymer that present excellent mechanical properties, chemical stability, biocompatibility, non-toxicity, and excellent film-forming capacity^51^. We utilized polyimide (Kapton) and high-purity copper as substrates, along with anisole as the solvent, to provide flexibility, durability, and optimal film formation for neutron detectors. Polyimide substrates have much smoother surfaces than metal sheets. However, the printed surfaces then need to be conductively coupled to the anode or cathode to avoid charge buildup.

**Fig. 1:**
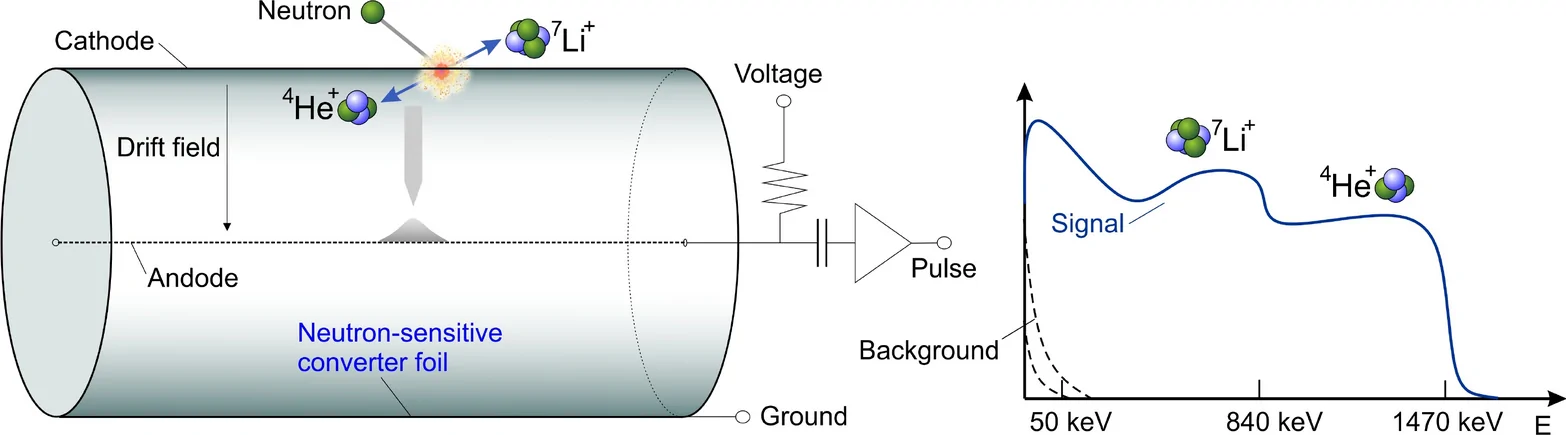
Schematic of a boron-lined tube (left) and pulse height spectrum (right). The neutron is converted into a helium and a lithium ion, which are emitted back-to-back. The primary ionization electrons drift towards the central anode with gas amplification near the wire. The pulse height spectrum is comprised of the full ionization peak and a plateau region of partial energy deposition below due energy loss within the conversion layer.

**Table 1:** Overview: neutron converters and their by-products of the main decay channels with kinetic energy and thermal absorption cross section at 25.2 meV (CS)^52^.

	Reaction		CS
$$^{6}$$Li	$$^{6}$$Li$$+ \text {n} \rightarrow $$	$$ ^{3}$$H+He*+4.78* MeV	940 b
$$^{10}$$B	$$^{10}$$B$$+ \text {n} \rightarrow $$	$$ ^{7}$$Li+He*+2.79* MeV (6.4 %)	3,837 b
	$$^{10}$$B$$+ \text {n} \rightarrow $$	$$ ^{7}$$Li*+γ +*He*+2.31* MeV (93.6 %)	

Although boron is a light element, its abundance is low compared with similar elements because the stable isotopes $^{10}$B (19.7±0.7 %) and $^{11}$B (80.1±0.7 %) are not part of the main solar fusion cycles. Enriched boron-10 is a by-product of the production of depleted boron, for example used in radiation-hard semiconductors. Boron nitride and boron carbide are among the hardest known materials after diamond, which adds complexity to fabrication methods based on sputtering^53^.

Figure 1 shows the schematic of a boron-lined proportional counter and its pulse height reaction product spectrum. Additionally, at the lower end there is a continuum of electronic noise and background events by photons. The low-energy contribution scales by the *γ * abundance and the volume to surface ratio of the device as part of this background originates from Compton electrons. Of relevance for low count rate applications is the contamination of the tube material by *α *-emitters^54^, which can be, dependent on the composition, in the order of 10$^{-4}$ cm$^{-2}$s$^{-1}$^55^.

This work presents the feasibility of printed neutron converter layers for CRNS and related detectors using functional printing. We fabricated boron carbide (B$_4$C) and lithium fluoride (LiF) films with polymer binders (PMMA, PVA) by blade coating on copper and polyimide substrates. We characterized their morphology, composition, thickness and roughness, stability and neutron response. We developed an automated SRIM-based workflow (MARS) to simulate conversion-ion energy loss and to interpret pulse-height spectra and detection efficiency versus layer thickness and binder content. We estimated scattering and absorption losses from binders and substrates at thermal energies to quantify their impact on detector performance. Lastly, we validated the printed films by measuring conversion spectra in proportional-counter tubes and compared them with sputtered references.

Methods first describes materials, printing, simulation workflow, and measurement setup. Results presents simulated converter-layer spectra/efficiencies, loss estimates, surface or elemental and film characterizations, concluding with the measured neutron conversion spectra.

## Methods

### Converter layer simulation

**Fig. 2:**
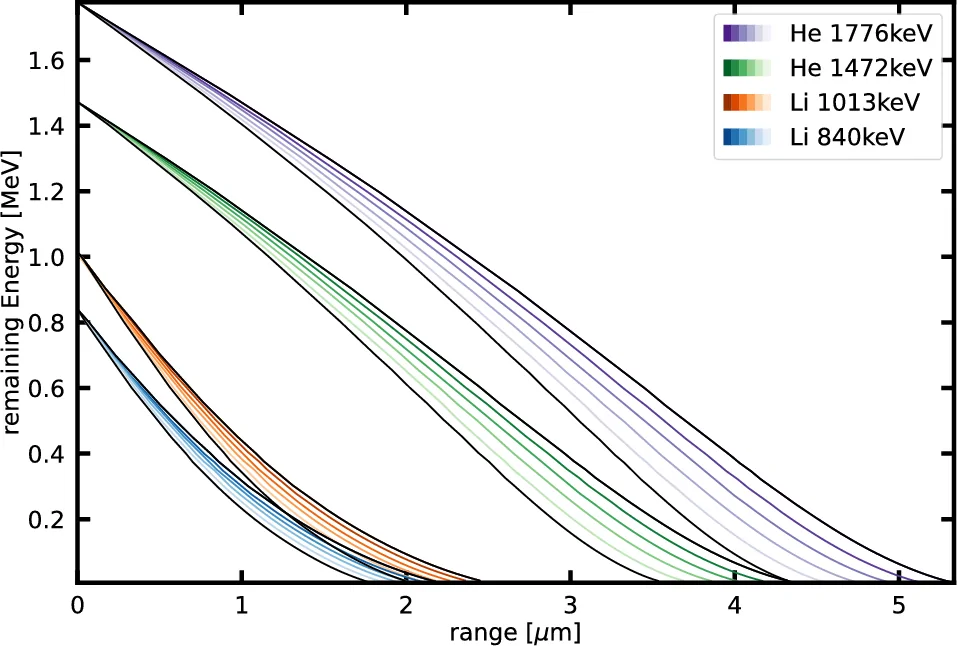
Distance-energy relation of the four possible conversion ions originating from the $\text {B}_4\text {C:PMMA}$ mixture with mixing ratios from 70 % to 95 % (lighter colors represent higher mixture ratios). Purple and orange belong to the 1776 keV + 1013 keV decay channel, green and blue to the main 1472 keV + 840 keV channel.

**Fig. 3:**
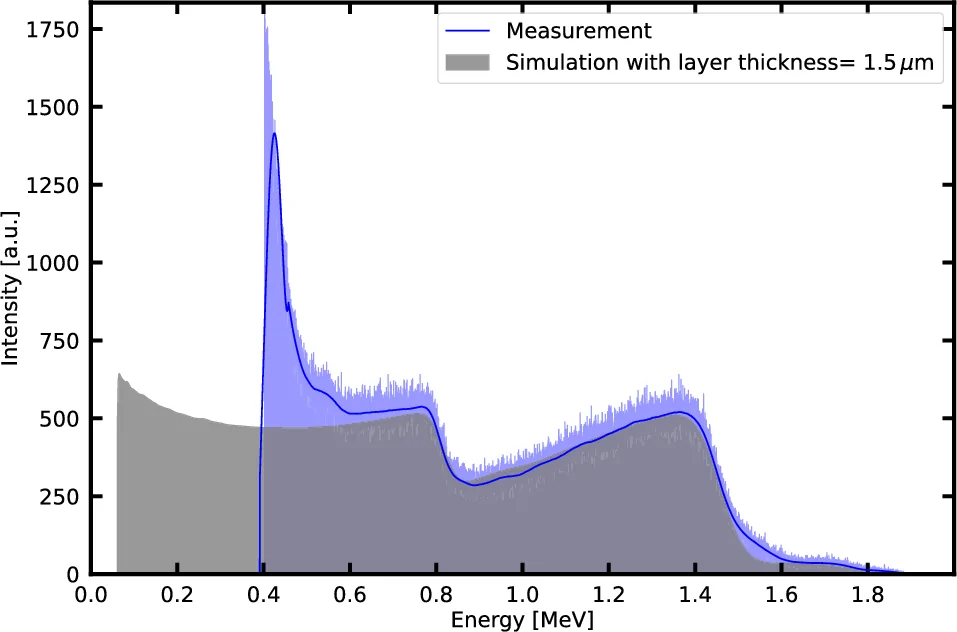
Exemplary comparison of scaled simulation and measured B$_4$C spectrum. Chosen simulation parameters: Non-ionizing zone = 1 nm, cutoff angle: 87$^\circ$, layer thickness: $\sigma =1.5\,$µm, Gaussian thickness variation: $\sigma =0.6\,$µm, Gaussian convolution: 7%.

The neutron pulse height spectrum can be used to assess average characteristics of a conversion layer^56,57^. For thin layers their thickness can be derived from such measurements significantly better than mechanical or x-ray-based methods. Therefore it is key to precisely understand the spectral shape. For neutron detector applications pulse height spectra calculations had been mainly done on the basis of time-consuming Monte-Carlo simulations in combination with stopping range tables^58,59^. In this work, we solved this problem by automating the creation of SRIM^60^ input files for materials with varying stoichiometry and mixture density. The resulting script is called MARS - Material Analysis for Radiation Spectroscopy^1^.

To derive the energy distribution of the escaping ions from different thicknesses of the material, the first step is to compute the energy loss over a traveled distance within this material d*E*/d*x* for a set of weight mixing ratios. The resulting SRIM calculations are stepwise travel distance tables for a given energy, which are then reversely integrated to simulate a particle with decreasing energy along its path. This intermediate step is shown in the pony tail plot in Fig. 2. As the range-energy curves do not overlap, it is possible to fit a family of functions for each ion depending on the mixing ratio. A polynomial fit of fourth order can be considered sufficient.

Each weight mixing ratio *k* was fitted by a fourth degree polynomial

1 $$\begin{aligned} f_{\text {ion,k}}(x)=a_{0,\text {ion}}x^4 + a_{1,\text {ion}}x^3 + a_{2,\text {ion}}x^2 + a_{3,\text {ion}}x \end{aligned}$$

and for the case of multiple mixing ratios, we created a family of fitting functions

2 $$\begin{aligned} f_{\text {ion}}(x, k)=\sum \limits _{i=1}^{4}f_{\text {ion,} k}(k)\cdot x^i. \end{aligned}$$

The numerical results for selected mixing rations are shown in Table 2.

**Table 2:** Stopping ranges of ions in $^{10}$B$_4$C:PMMA (left) and $^6$LiF:PMMA (right) mixtures at varying weight mixing ratios.

$^{10}$B$_4$C:PMMA	Range [µm] at mixing ratio				$^6$LiF:PMMA	Range [µm] at mixing ratio			
Ion	50%	75%	90%	100%	Ion	50%	75%	90%	100%
He 1,776 keV	6.15	5.21	4.6	3.97	$^3$H 2,734 keV	45.5	40.03	36.31	33.29
He 1,472 keV	5.16	4.33	3.79	3.24	He 2,051 keV	8.07	7.28	6.65	5.94
Li 1,013 keV	2.92	2.44	2.18	2.13					
Li 840 keV	2.61	2.18	1.93	1.88					

Then, the onedimensional curves are mapped to a threedimensional layer geometry. In this work, we treated the material as infinitely extended in two directions with a thickness *t*. This allows the ions to escape under any angle in the direction of the detector. The longitudinal distribution of conversion ion pairs follows a double-sided exponentially decreasing probability, depending on the cross section of the target element, as well as weight of the material. The resulting distance distribution probability is constant until the path length *x* reaches *t* and consecutively decreases by $\approx 1/x^2$. Given the distance distribution the spectral energy distributions in the counting gas can then be computed for each material thickness and mixing ratio. To compare this theoretical approach with measurements, we applied a Gaussian convolution (7 % FWHM) reflecting the characteristics of the detector and front-end electronics used in this work. Figure 3 shows an exemplary comparison of such a simulated pulse height distribution in comparison to a measured spectrum (with a gamma-induced cutoff at 0.4 MeV related to the measurement at the neutron source). Following, the efficiency can then be calculated using tools such as DECal^61^.

### Sample preparation

**Fig. 4:**
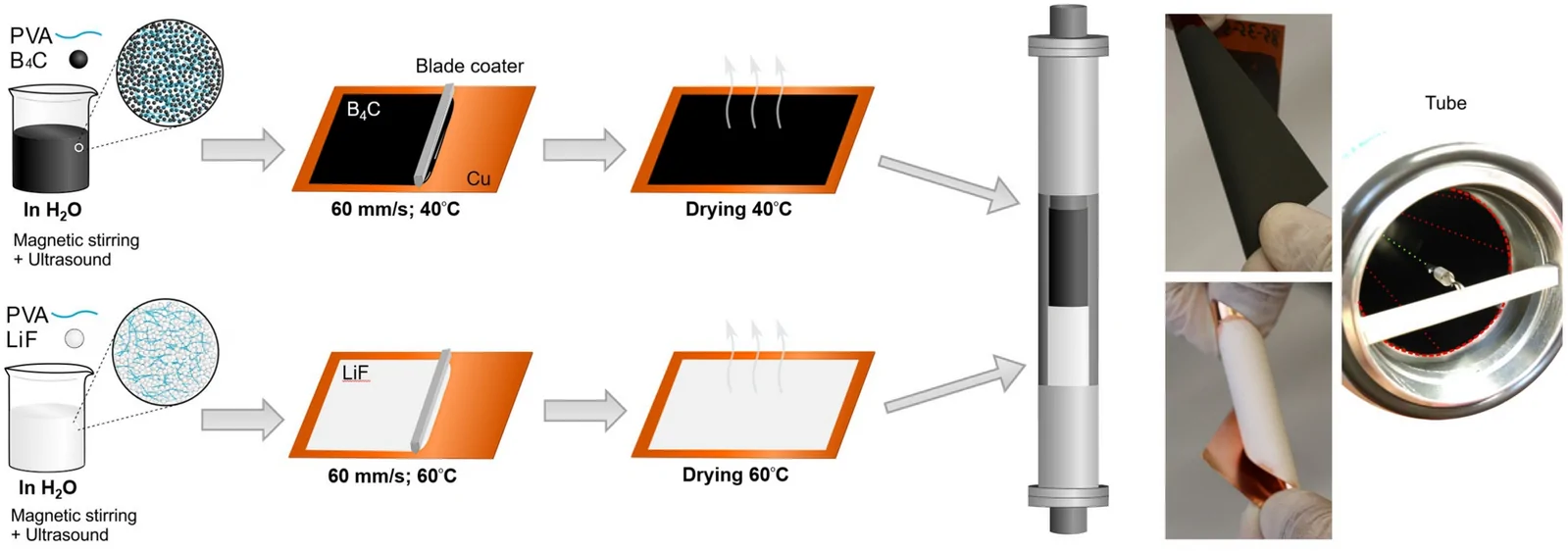
Scheme representing the sample preparation for both thin film materials. The solutions were mixed and then distributed evenly with the blade coater. The solvent is then evaporated on a hot plate. The foils are rolled into a counting tube, which is then vacuum-baked for 16–24 hours at approximately 80$^\circ$C.

Two mixtures were prepared through simple mixing in a solvent dispersion. PMMA (Molecular weight 15,000 g/mol, Acros Organics) was dissolved in anisole (Sigma-Aldrich) at a concentration of 31.7 mg/mL stirred magnetically at an optimized speed of 900 rpm for 30–45 min at room temperature. PVA (Molecular weight 20,000 g/mol, 98 % hydrolyzed, Sigma-Aldrich) was dissolved at a concentration of 44 mg/mL in deionized water at 90$^\circ$C by the same method. Additionally, 15 min resting time in an ultrasonic bath forced gas bubbles out. We then added the filler at the chosen weight percentage relative to the binder. The mixture was kept on the magnetic stirrer under the same conditions for another 30–45 min, placed in the ultrasonic bath for 1–2 h, and finally stirred again for 10 min. Sonication over an extended period is a crucial step for the suspension processing technique as the high-frequency sound waves break down the agglomerates of the active material to ensure its uniform distribution in the solution^62^. We cleaned the substrate with deionized water and isopropanol (IPA) and then exposed it to a nitrogen stream to remove any remaining dust particles. The hot plate of the blade coating machine was heated to the working temperature: 40$^\circ$C for B$_4$C (0.8 µm grain size, 3M) and B (*≤ *5 µm grain size, 3M) or 60$^\circ$C for LiF (Sigma-Aldrich). Then, the formulated dispersion was poured on one size of the substrate using a pipette. By linear movement of a blade coater (Erichsen) at speeds of 10 mm/s to 60 mm/s the suspension was distributed over the substrate to produce a homogeneous film. After the coating process the heat supplied through the hot plate evaporates the solvent. The steps are shown in Fig. 4 (left) with application examples (right).

### Sample film characterization

**Fig. 5:**
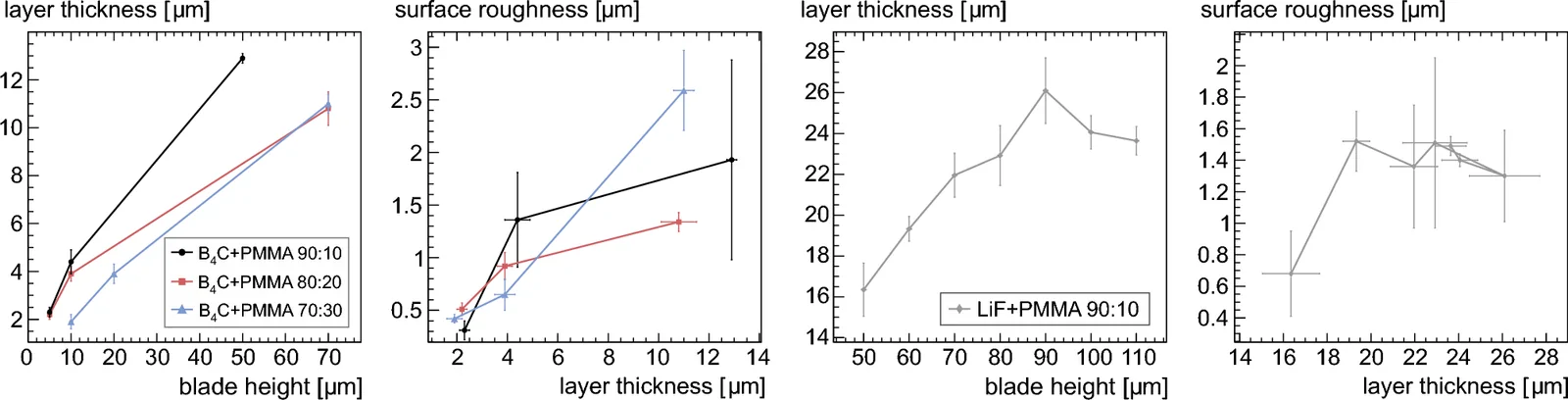
Film topology characterization for the samples B$_4$C + PMMA (left) and LiF + PMMA (right) with respect to layer thickness and surface roughness. Error bars indicate typcial variations on a single sample.

The blade height of the coater was varied between 5 µm and 110 µm to yield different thicknesses of the layers after solvent evaporation. The coating speed was varied between 10 mm/s and 27 mm/s for B$_4$C and LiF with 15 mm/s achieving the best results in terms of uniformity and roughness. The film thickness was determined by locally wiping the film with a cotton swab and measuring the resulting step height with a mechanical profilometer (Veeco). Figure 5 shows the relation of the film thickness and roughness of B$_4$C films as a function of blade height. It can be observed that the minimum adjustable blade height yields B$_4$C films with a thickness on the order of 2 µm. Processing thin films involves several mechanical steps, therefore the layer surface roughness is higher than can be achieved for sputter deposition. The achievable roughness and layer height are also a factor of the amount of solvent. For B$_4$C batches the anisole concentration was varied between 200 mg/mL and 550 mg/mL and for LiF setups between 500 mg/mL and 800 mg/mL. A higher solvent concentration generally yields thinner films due to increased solution spreading leading to a thin film after drying. Lower solvent surcharges yield more thick formations and allow drying with less heat and standing time for the evaporation of the solvent.

### Neutron measurements

The solution-processed layers were evaluated with respect to their signal characteristics at the californium source at the Physikalisches Institut, Heidelberg University. The lab-scale neutron source has two experimental stations. Its spectrum is undermoderated, resulting in a main thermal component and a secondary epithermal component^63^. We recorded the characteristics directly at the beam port of the neutron source, which has a width of 10 cm and a height of 5 cm. It leads to a count rate of around 600 Hz for counting tubes with enriched boron or lithium. Tubes equipped with natural boron carbide converters yield a rate of slightly above 100 Hz. We rolled the prepared samples, placed them into proportional-counter tubes (see Fig. 4), vacuum-baked them, and filled them with Ar:CO$_2$ (90:10) at 300 mbar. The tube design^15^ and front-end electronics^64^ are our own designs. The resolution was approximately 7 % FWHM. Signal heights and rates were recorded by a Red Pitaya STEMlab 125-14 multi-channel analyzer.

## Results

### Converter layer spectra and efficiencies

**Fig. 6:**
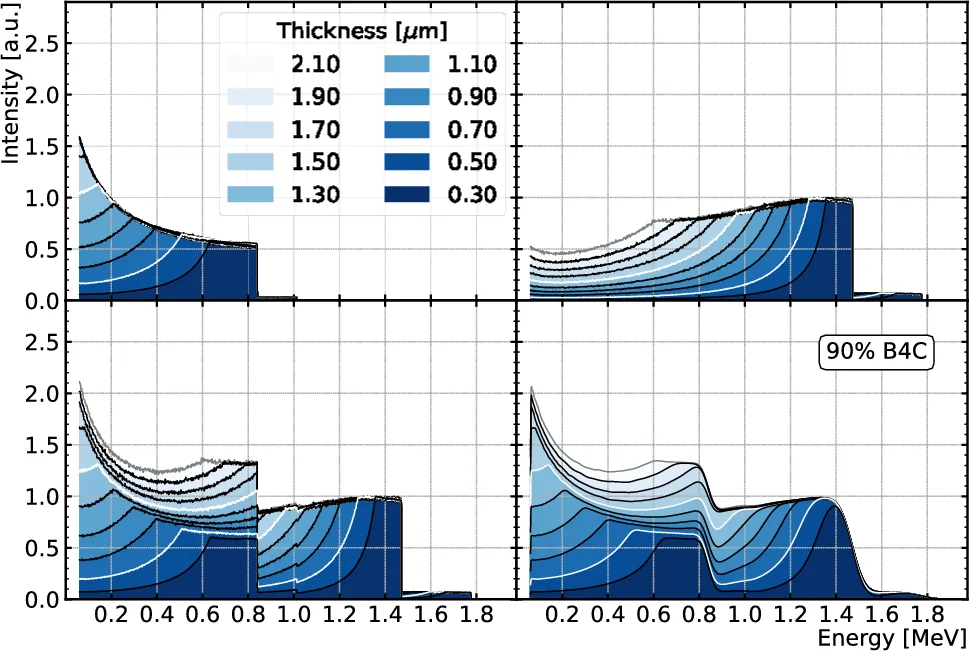
Simulated neutron conversion energy distribution of a 90 %$_\text {wt}$ $^{10}\text {B}_4\text {C:PMMA}$ mixture with varying material thickness. Top: energy deposition spectra of lithium (left) and helium ions (right), bottom left: combined energy distribution, bottom right: combined energy distribution with 7% FWHM Gaussian convolution.

In order to evaluate the effects of different layer structures, the signal yields from the conversion ions need to be interpreted. Figure 6 provides an overview of how the simulated spectra as measured in a detector change with layer thickness. A result of using boron-lined converters is a spectrum which continuously ranges from the maximum conversion energy given by the *q*-value, see Table 1, to zero. The reason is that ions traversing through the absorber material experience a significant energy loss. The remaining energy when entering the counting gas can span the full range of potential energies - originating from conversions right on the surface to those ’deep’ inside the material. Lithium ions with their lower initial energy, larger mass and charge are stopped more easily, therefore their range is approximately only half of their helium counterpart^65^.

**Fig. 7:**
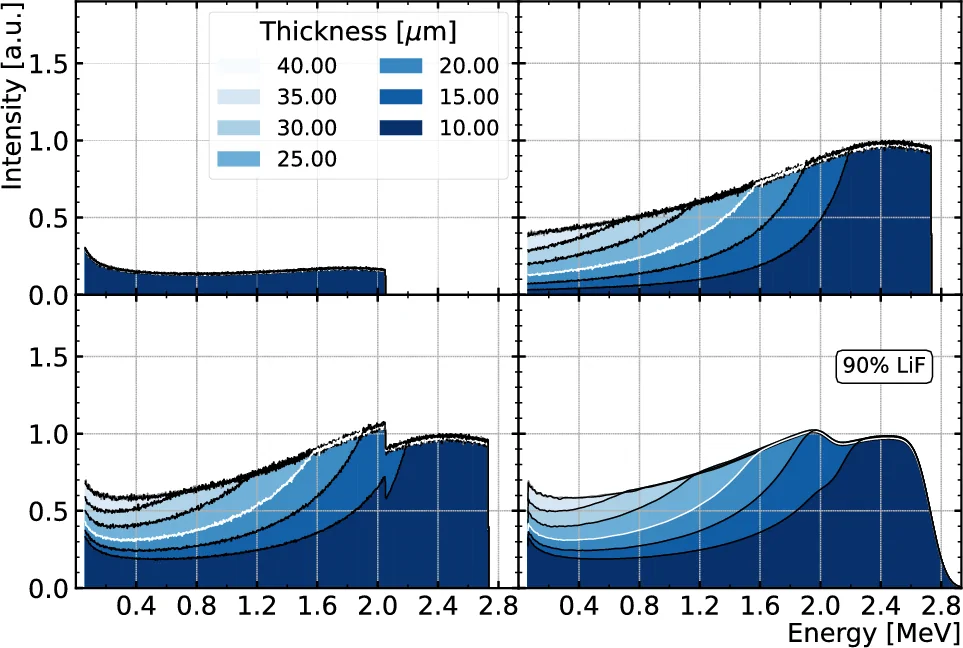
Neutron energy distribution of a 90 %$_\text {wt}$ $^{10}\text {LiF:PMMA}$ mixture with varying layer thickness. Top: energy spectra of helium ions (left) and tritium (right), bottom left: combined energy distribution, bottom right: combined energy distribution with 7% FWHM Gaussian convolution.

Larger converters will inevitably have a larger zone of inactive volume where particles are less likely to escape. For the lithium ions there is effectively no gain in efficiency any more beyond 2 µm of boron carbide layer height, see Figs. 2 and 6. Whereas helium ions at such a thickness would still contribute there is a growing pileup for the lowest detectable energies. The difference between absorption probability of a thermal neutron and the signal detection efficiency is shown in Table 3. In an actual instrument a threshold of 50 keV to 100 keV would need to be set in order to reject contributions from particles other than neutrons. A convenient layer thickness lies around 1.5 µm considering a tradeoff of base material used for the film and detection efficiency, see also^66^. Compared to other possible elements boron and lithium have a high stopping power for ions. The negative impact of adding carbohydrates as a binder and therefore inactive volume is partially compensated by the better ion transparency of the mixture. Adding 10 % of PMMA by weight to a boron carbide film increases the range of the helium ions by approximately 16 % and for the lithium ions by around 3 %. Adding 25 % of PMMA increases the helium range by 30 % and 50 % of PMMA around 55 %. For lithium ions the transparency of the mixtures is much less emphasized. The reason is that in terms of energy loss they begin their trajectory already beyond the Bragg peak and therefore are subject mainly to terminating stopping effects. Helium ions have a lower mass and are ejected with higher energies, leading to them starting before the Bragg peak and having a ’buffer’ of usual d*E*/d*x* interactions.

For lithium fluoride, see Fig. 7, a similar behavior can be observed. The total kinetic energy of the conversion products is larger, hence, both ions start well before the Bragg peak. As a result of the energy deposition process and the lower stopping power of lithium fluoride much thicker layers can be achieved. As lithium-6 has a lower absorption cross section compared to boron, such thicker layers are on the other hand required to achieve the same efficiency. In order to understand the performance of both materials, the interplay between cross section, energy loss in the layer and the effect of adding a binder needs to be described precisely.

**Table 3:** Comparison of different converter materials (C) and thicknesses (d [µm]) with the total thermal neutron absorption probability (A). The ions (I) reaching the gas [%] without threshold/50 keV/100 keV threshold yield a specific layer efficiency [%] *ε * (absorption *× * ion efficiency). The efficiency [%] if used as a converter in a proportional counter tube in a CRNS detector (D$_\epsilon$) is calculated with the full neutron spectrum and a threshold of 100 keV.

$^6$LiF					$^{10}$B$_4$C				
d	A	I	*ε *	D$_\epsilon$	d	A	I	*ε *	D$_\epsilon$
10.4	5.8	58.1/56.7/55.9	3.3/3.2/3.2	3.86	0.9	3.87	81/77/74	3.13/2.98/2.86	3.77
15.6	8.6	48.9/47.7/47.1	4.2/4.1/4.1	4.24	1.1	4.72	77/72/69	3.63/3.40/3.26	4.14
18.2	10.0	45.7/44.6/44.1	4.6/4.5/4.4	4.41	1.3	5.55	73/67/64	4.05/3.72/3.52	4.25
19.2	10.5	44.6/43.6/43.0	4.7/4.6/4.5	4.39	1.5	6.38	69/63/59	4.4/4.02/3.76	4.31
20.8	11.3	43.4/42.4/41.8	4.9/4.8/4.8	4.43	1.7	7.19	64/58/55	4.6/4.17/3.95	4.38
23.4	12.6	40.4/39.4/38.8	5.1/5.0/4.9	4.38	1.9	8.00	60/54/51	4.8/4.32/4.08	4.39
31.2	16.5	33.3/32.4/31.9	5.7/5.5/5.5	4.15	2.1	8.81	56/51/48	4.93/4.49/4.23	4.37
41.6	21.3	24.7/23.9/23.5	5.3/5.1/5.0	3.45	2.3	9.60	53/48/45	5.10/4.60/4.30	4.28
					2.5	10.4	50/45/42	5.20/4.70/4.40	4.16
					3.0	12.3	43/39/36	5.30/4.80/4.40	4.03

Such a performance summary is presented in Table 3 for different layer thicknesses. As a result, the partial loss of detection efficiency can nearly be compensated by a larger layer thickness. On average the efficiency losses from adding binder materials are low as the binder is rather transparent for neutrons and its stopping power for ions is low. Another result is that possible trace contaminations of heavy metals originating from boron-carbide processing have a negligible effect. Their presence does not noticeably alter the detector response, since the neutron converter layers already a considerably high stopping power. Results are shown in Table 4 with an exemplary tungsten impurity as could be a result of ball milling. In summary, the negative effect on the total efficiency of adding non-converter materials to a boron carbide or lithium layer is rather small.

**Table 4:** Influence of the binder and tungsten contamination for boron carbide layers of 1.5 µm thickness.

Detection probability for	He ions [%]	Li ions [%]
$^{10}$B$_4$C	39.7	29.0
$^{10}$B$_4$C + 10 % PVA	40.3	29.7
$^{10}$B$_4$C + 10 % PMMA	39.5	29.8
$^{10}$B$_4$C + 10 % PVA + 0.5 % W	40.4	30.0

Finally, when considering a detector with a polyethylene housing, such as a CRNS instrument, it must be taken into account that neutrons undergo multiple scattering events inside the moderator^10^ and that a (low) energy threshold is applied. That introduces another optimization variable. For multiple detector passes the inactive volume negatively affects the total detection probability as absorbed undetected neutrons are not available for the following pass. Hence, as calculations show, there is an optimum layer thickness of around 20 µm for lithium fluoride and 1.7-2.1 µm for boron carbide films.

### Calculation of neutron losses by scattering and absorption

Analyzing the neutron scattering background caused by the detector itself^67^ or its components^68,69^ is crucial because it can significantly distort experimental data, leading to incorrect interpretations of data and signal dynamics. Additionally, absorption losses in non-active detector materials (e.g., housing, windows, or shielding) reduce neutron detection efficiency and must be accounted for to arrive at an accurate flux normalization or intensity corrections. Understanding these effects is a relevant factor in instrument design and allows more reliable quantitative analysis in neutron scattering experiments. In applications of neutron detectors with neither strict requirements on position nor time the scattering background is of less importance than the self-absorption. For large-scale research facilities or fundamental physics applications scattering contributions need to be taken into account. Adding a hydrogen-rich binder, even in the low concentrations required for printing, can be a source of absorption and scattering. Therefore, we estimated signal losses at thermal neutron energies (25 meV) for the fabricated films and the binder, and we compared them with sputter-deposited layers and substrate materials. The macroscopic cross sections for elastic scattering ($\Sigma _{\text {scat}}$) and absorption ($\Sigma _{\text {abs}}$) were determined by combining the known thermal cross sections $\sigma _{\text {th}}$ with the atomic densities *N* via $\Sigma _{\text {th}} = N \sigma _{\text {th}}$. For this estimation for elastic scattering the coherent and incoherent cross sections were summed up. The thermal neutron losses due to elastic scattering and absorption were expressed as percentages:

3 $$\begin{aligned} \text {Loss} = (1-p_T) \cdot 100 \quad \text {with}\quad p_T = e^{-\Sigma _{\text {th}} d}. \end{aligned}$$

with the transmission probabilities $p_T$ for a given thickness *d*. The results are summarized in Table 5 for different materials and exemplary thicknesses which could be found in neutron detectors.

**Table 5:** Approximated neutron scattering and absorption losses (in %) for the active layer (left) and in comparison to the substrate materials aluminum (AlMg3), oxygen-free copper (OFC), stainless steel 304L and Kapton (polyimide) (right). Absorption losses for boron carbide include the desired neutron detection.

Layer	Thickness	Scatt. Loss	Abs. Loss	Substrates	Thickness	Scatt. Loss	Abs. Loss
Boron carbide (B$_4$C)	2 µm	0.01 %	8.24 %	Aluminum	0.1 mm	0.35 %	0.05 %
B$_4$C + 10 % PVA	2 µm	0.03 %	6.85 %	Aluminum	4 mm	13.12 %	1.96 %
B$_4$C + 10 % PVA	2.42 µm	0.03 %	8.24 %	Copper	50 µm	0.35 %	0.16 %
PMMA binder (10 %$_\text {wt}$)	2.42 µm	0.02 %	0 %	Stainless steel	0.1 mm	1.57 %	0.47 %
PVA binder (10 %$_\text {wt}$)	2.42 µm	0.02 %	0 %	Stainless steel	2 mm	27.19 %	9.08 %
PVA binder (30 %$_\text {wt}$)	2.42 µm	0.07 %	0 %	Kapton	50 µm	1.10 %	0.01 %

As a result the contribution of the binder to potentially adding concerning scattering or absorption reactions can be considered negligible. Any potential substrate has a significantly larger influence to the detector characteristics than the additional contribution from the binder.

### Surface characterization

**Fig. 8:**
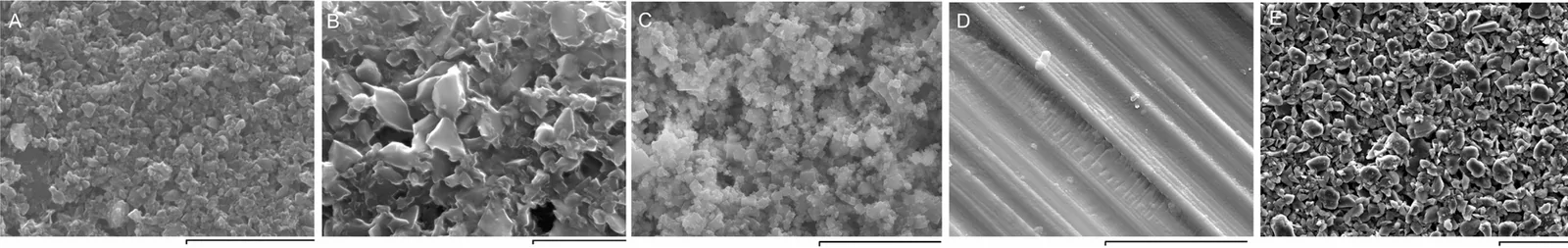
Scanning electron microscope images of neutron converter layers on a copper substrate: (**A**) boron carbide with PMMA in 10 µm scale (black bar below the image), (**B**) boron carbide with PVA in 2.5 µm scale, (**C**) lithium fluoride with PVA in 10 µm scale, (**D**) with sputter-deposited boron carbide in 10 µm scale and (**E**) boron metal with PVA in 50 µm scale. The images show the morphology of the converter, the binder is mostly not visible in these measurements.

The morphology and microstructure of the neutron converter layers deposited on a copper substrate were investigated using scanning electron microscopy (SEM), see Fig. 8. The SEM analysis reveals the impact of material composition and deposition methods on the structural properties of neutron converter layers, which are critical for their performance in neutron detection applications. The images of the samples show distinct surface features and structural variations among the different material compositions and deposition methods:

Boron carbide with PMMA exhibits a homogeneous distribution of particles at the 10 µm scale, with minimal agglomeration and a smooth surface morphology, indicative of effective polymer integration. On the 2.5 µm scale (Sample B with PVA) a more porous and interconnected layering of the boron carbide flakes can be found. Lithium fluoride with PVA shows a uniform granular morphology at the 10 µm scale, with well-defined particle boundaries of the cubic-shaped crystals and a compact arrangement, suggesting favorable adhesion and coverage on the substrate. As a comparison, the sputter-deposited boron carbide (reference sample) presents a dense and continuous layer with a notably smoother and more uniform surface compared to the solution-processed counterparts. The observed more coarse morphology and porosity might be beneficial for increasing the detection efficiency due to the surface enlargement^70,71^.

### Elemental analysis

**Fig. 9:**
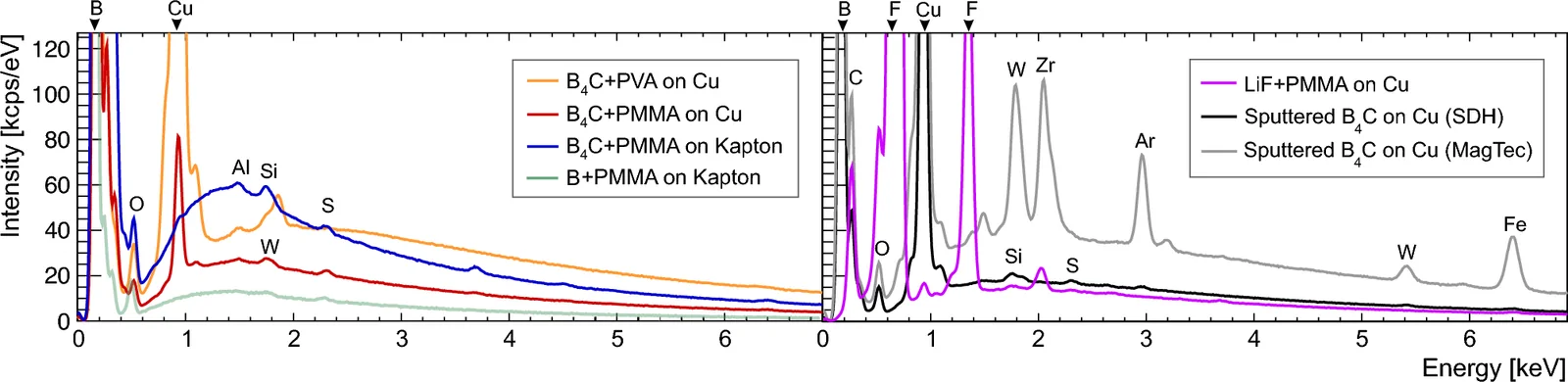
Energy-dispersive X-ray spectroscopy analysis of the films with boron carbide, boron and lithium fluoride in comparison to sputter deposited layers (right).

To assess the elemental composition and potential impurities in the neutron converter layers, energy-dispersive X-ray spectroscopy (EDS) was employed in conjunction with scanning electron microscopy. EDS provides spatially resolved elemental mapping and quantitative analysis, enabling the identification of characteristic X-ray peaks associated with the constituent materials. Given the layered nature of the films, particular attention was paid to distinguishing between intended converter materials, substrate contributions and possible processing-induced contaminants. An overview is presented in Fig. 9 and Table 6.

Figure 9 shows the EDS analysis of the fabricated films and compares it to two sputter-deposited boron carbide samples from MagTec GmbH, Germany, and S-DH GmbH, Germany. The intensity of the peaks and the background depend on the layer thickness. The contribution from the main elements like boron, carbon, fluorine and copper was cut. Table 6 summarizes the results from the elemental analysis. Tungsten or zirconium can be present as a result from milling boron carbide or recycling sputter targets. In one of the sputter-coated samples (MagTec GmbH) this contamination was approximately 20 %. This was too high to serve as a reference quantification and was not shown in Table 6. Yet, traces of tungsten or zirconium can be found in the other films as well. One notable contribution is oxygen which is present in all samples as part of the binder. Sputtered coatings on the other hand have a large number of lattice defects which leads to oxygen integration on the surface, partially diffusing deeper. Other measurements (not shown) had revealed hydrogen diffuses into the sputtered layers. Yet, in EDS it cannot be resolved. The solution-processed layers show a very low contamination of unwanted elements. Especially the boron + PMMA sample show a very high purity with no clearly visible elements other than those expected from the film composition. As a result we can conclude that solution-processed neutron converters have the potential to create high-purity layers with reliable quality.

**Table 6:** Elemental (Elm) composition (mass %) of: (1) B$_4$C+PVA on Cu, (2) LiF+PMMA on Cu, (3) B$_4$C+PMMA on Kapton, (4) B$_4$C+PMMA on Cu, (5) Sputtered B$_4$C on Cu (SDH). The Cu substrate was removed from the analysis. Boron B and carbon C intensities are combined. Uncertainties in parentheses represent one standard deviation (0 if smaller than the last digit).

Elm	(1)	(2)	(3)	(4)	(5)	Elm	(1)	(2)	(3)	(4)	(5)
B+C	95.85(3)	–	97.87(6)	96.46(8)	93.74(7)	Na	–	0.33(1)	–	–	–
F	–	98.09(2)	–	–	–	Mg	–	0.17(1)	–	–	–
O	2.26(1)	1.06(1)	1.01(1)	0.90(2)	6.10(3)	Si	–	0.06(0)	–	0.04(0)	–
N	–	–	0.91(6)	2.45(8)	1.20(5)	Ca	–	0.05(0)	0.05(0)	0.03(0)	–
W	1.83(3)	–	0.01(2)	–	2.29(5)	Ti	–	–	0.03(0)	0.02(0)	–
Fe	0.03(1)	0.12(1)	0.06(0)	0.06(1)	0.14(1)	Cr	–	–	–	–	0.09(1)
Al	0.03(0)	–	0.02(0)	–	–	Ar	–	–	–	–	0.06(1)

### Outgassing

A major concern for gaseous detectors is the slow evaporation of molecules which lead to a degradation of the sensor performance^72^. Ion- and electron-attaching (electronegative) gases like water or oxygen have the potential to reduce the gas gain. Uncontrolled hydrocarbons polymerize on electrodes^73^, causing aging^74^. Specifically in hermetically sealed proportional counters the selection of suitable materials is critical^75^. Gases like the popular Ar:CO$_2$ are rather tolerant to impurities, however, already 50 ppm of oxygen can significantly lower the amplification. For the solution-processed converter layers, several concerns arise: the surfaces may not be mechanically stable enough for wipe-cleaning (risking residuals from handling), moisture could be trapped on the rough surface and may not evaporate during baking, and, most importantly, the binder or solvent remnants could outgas. We conducted long-term tests with hermetically sealed tubes and measured the position of the inflection point of the 1,472 keV ion for boron carbide or the peak position for lithium fluoride. Within the currently running project period B$_4$C+PVA films did not show any degradation on a timescale of 19 months, B$_4$C+PMMA films were up to now stable for 16 months and LiF+PMMA layers for 19 months.

Within the first month the spectrum shifted slightly upwards or downwards, which is usual, and then stabilizes. Usual effects from gas contaminations or plasticizers of sealing rings lead to a degradation of 0.1 % to 1 % in 10 days. Neither the lithium fluoride nor the boron carbide samples in combination with PVA or PMMA have shown any measurable degradation. However, on samples which were produced from ball-milled boron carbide, degradation effects were found, which is currently under investigation. The assumption is that due to the much smaller sized particles significantly longer baking times would be necessary. As a conclusion, currently there is no evidence for major long-term outgassing effects of the binder.

### Neutron characterization

**Fig. 10:**
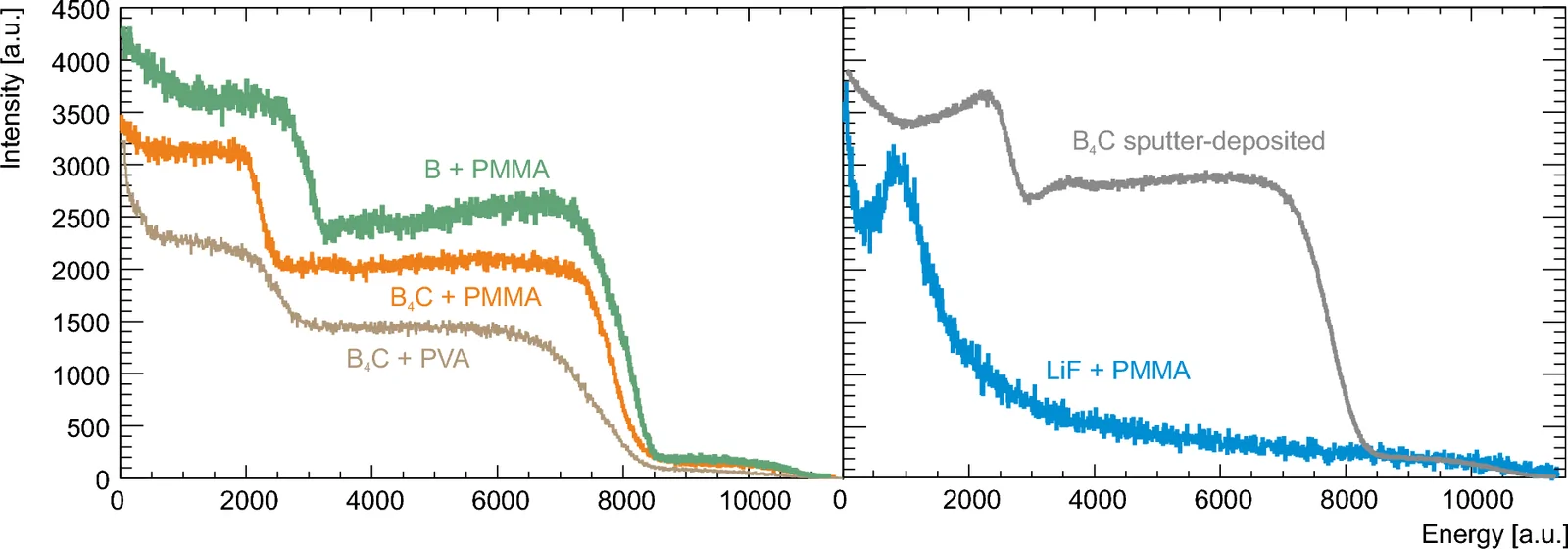
Measured spectra with converter foils mounted in proportional counter tubes (length 1250 mm, diameter 54 mm, Ar:CO$_2$ 90:10, 300 mbar). The gain is not uniform and spectra are normalized to each other. The sharpness of the edges is a measure of layer thickness variation. Thickness of the layers: B$_4$C+PMMA: 2.2 µm, B$_4$C+PVA: 3 µm, B+PMMA: 2 µm, sputtered 1.5 µm. Lithium fluoride + PVA: 30 µm.

The quality of the layers is evaluated by measuring the neutron conversion ion spectrum. Inhomogeneities, potential inactive surface layers or impurities would change the shape with respect to a reference spectrum. Figure 10 presents a selected comparison of four different samples with layer thicknesses *≥ 1* µm. The sputter-deposited boron carbide layer acts as reference sample and shows the most sharp edges. The boron carbide film with PMMA shows a comparable quality. The boron carbide and PVA sample from the first batch shows more smeared out edges. This is a result of layer thickness variation and surface roughness. Although the samples are aimed to achieve a thickness of 1.5 µm, the blade coating setup does not support such a fine scaling. Lower converter layer heights were achieved by variation of the solvent content. In the case of the B$_4$C plus PVA sample, already optically inhomogeneities could be clearly identified. The lithium fluoride with PVA film, not to scale with the boron samples, exhibits a clearly distinguishable peak. Even at ambient gas pressure, the proportional-counter tube diameter is not large enough and the much longer tracks of the lithium conversion ions are geometrically cut and stopped at the opposite side. Given that, the spectral shape for LiF presents itself as expected.

The achieved neutron detection efficiency varies with coating quality. The current findings indicate that a more rough surface leads to a lower overall neutron yield. The ranged of measured neutron rates within a detector setup varied around from 50 % to above 90 % (most coarse grained to fine grained) compared to a sputter-coated foil of equally high thickness.

### Cost assessment

To compare proportional-counter–based CRNS options on cost, we normalize across detector types to deliver approximately the same count rate under typical conditions. Because converter media differ in absorption cross section and practical thickness, we use commercially representative tube geometries that achieve similar performance rather than identical dimensions. For comparing the costs per area the relative efficiencies for a proportional counter tube are presented. Costs are per proportional counter (detector tube) and exclude frontend electronics and housing. The normalization target is a similar CRNS count rate, dimensions chosen accordingly (Table 7).

**Table 7:** Preliminary cost estimation for CRNS detector tubes normalized to comparable count rates. Tube geometries chosen to yield approximately equal count rates in a CRNS system. For ^3^He, the cost is dominated by the gas filling, for BF_3_ (hazardous), around 1000 € per tube plus the gas filling, for other types, base tube *≈ *500 € plus converter cost. Thermal absorption efficiencies refer to converter materials only. Printed-converter options sized to match the same count-rate class.

Metric	^3^He	^10^BF_3_	^10^B_4_C (sputtered)	^10^B_4_C (printed)	^6^LiF (printed)
Tube geometry	2 in *× * 12 in	2 in *× * 30 in	2.3 in *× * 47 in	sized to match	sized to match
Unit cost [€]	2500	1500	1200	700	900
Thermal absorption eff. [%]	60	20	12	11	12

## Conclusion

This study demonstrates the feasibility of fabricating cost-efficient neutron converter layers for Cosmic-Ray Neutron Sensing and other neutron detection applications using functional printing techniques. By optimizing the deposition of boron carbide (B$_4$C) and lithium fluoride (LiF) with polymer binders (PMMA, PVA), we achieved stable and efficient neutron converter films. Simulations confirmed that B$_4$C layers of approximately 1.5 µm and LiF layers of 20 µm provide an optimal trade-off between neutron absorption efficiency and ion escape probability. The addition of binders (PMMA/PVA) minimally impacts detection efficiency and characteristics. The blade-coating process proved itself suitable for cost-effective production, with SEM revealing uniform morphologies comparable to sputter-coated reference samples. Conversion spectrum measurements in proportional counter tubes validated the response of the solution-processed films, particularly B$_4$C+PMMA, which exhibited sharp edges comparable to conventional sputtered layers. Long-term stability tests showed no significant outgassing. The findings emphasize the importance of printed neutron converters as a scalable substitute for sputter-deposited layers, with potential uses in environmental monitoring, large-scale research facilities or portal monitors. Future research will concentrate on investigating improved ink-formulations and the use of other processing techniques such as screen printing which would enable the 2D-patterning of the deposited films as well as different materials.

## Data Availability

Data will be made available upon request.

## Footnotes

1 MARS is available at https://gitlab.com/stand1ngwave/mars
